# Breathing Interventions Improve Autonomic Function, Respiratory Efficiency and Stress in Dysfunctional Breathing: A Randomised Controlled Trial

**DOI:** 10.3390/arm93060056

**Published:** 2025-12-10

**Authors:** Laura Zaliene, Alvina Mockute, Lina Levickiene

**Affiliations:** Department of Rehabilitation and Aesthetic Therapy, Faculty of Health Sciences, Klaipedos valstybine kolegija/Higher Education Institution, LT-92255 Klaipėda, Lithuania; alv.mockute@kvkedu.lt (A.M.); l.levickiene@kvk.lt (L.L.)

**Keywords:** heart rate variability, breathing interventions, dysfunctional breathing, stress reduction, biofeedback

## Abstract

**Highlights:**

**What are the main findings?**

- A 6-week randomized controlled trial examined guided breathing with and without nocturnal mouth taping in women with dysfunctional breathing.
- Both groups showed significant reductions in respiratory rate and perceived stress after the breathing program.
- Only the mouth-taping group demonstrated significant improvements in the high-frequency component of HRV and Hencho breath-hold performance.
- The multi-component breathing intervention was safe, low-cost, and feasible in a community fitness setting.

**What is the implication of the main finding?**

- Findings support physiotherapy-led breathing interventions as a non-pharmacological strategy to enhance autonomic regulation and respiratory efficiency.

**Abstract:**

Background: Dysfunctional breathing patterns may impair autonomic regulation and increase perceived stress. Breathing-based interventions, particularly those involving guided exercises and supportive tools, have the potential to provide non-pharmacological benefits. Methods: In this parallel two-arm randomized controlled trial, 14 women aged 35–45 years with signs of dysfunctional breathing and no comorbidities were recruited from a fitness club. Participants were randomly assigned (1:1) using a computer-generated sequence to an intervention group (*n* = 7) or a control group (*n* = 7). Blinding was not applied. Both groups completed a 6-week program of guided breathing exercises using the iBreathe app, while the intervention group additionally used mouth tape during sleep. The primary outcomes were heart rate variability (HRV) indices—root mean square of successive differences (RMSSD) and the high-frequency (HF) component. Secondary outcomes included respiratory rate, Hencho test performance, and perceived stress measured using the Perceived Stress Scale-10 (PSS-10) and a Visual Analogue Scale (VAS). All participants were included in the final analysis (no loss to follow-up). Results: The intervention group showed a significant increase in the HF component of HRV (*p* = 0.018) and improved Hencho test performance (*p* = 0.018). Both groups demonstrated significant reductions in respiratory rate (*p* < 0.05) and PSS scores (*p* < 0.05). Between-group differences were not significant for RMSSD or perceived stress. No adverse events were reported. Conclusions: A 6-week breathing intervention improved respiratory efficiency and reduced perceived stress among women with dysfunctional breathing. The additional of night-time mouth taping provided further benefits for HRV and respiratory control. Larger and longer trials are needed to confirm these findings.

## 1. Introduction

Breathing is a fundamental physiological process ensuring that the body’s metabolic demands are met while also reflecting emotional and psychological states. Although breathing is primarily automatic, it can be consciously regulated, allowing individuals to influence both physical and mental health, as well as rehabilitation outcomes. In physiotherapy practice, dysfunctional breathing has become an important clinical focus, as it may compromise postural stability, movement control, and overall functional capacity. Dysfunctional breathing is characterized by inefficient patterns [1], such as excessively rapid, shallow, or mouth breathing [2], which can contribute to autonomic nervous system imbalance, heightened stress, and disturbances in homeostasis [3].

Heart rate variability (HRV) is a key marker of autonomic nervous system activity and reflects the body’s capacity to adapt to internal and external demands. Low HRV is associated with chronic stress, anxiety, burnout, and reduced resilience to disease [4], and has also been linked to poorer functional performance and slower rehabilitation progress. Dysfunctional breathing may adversely affect HRV by reducing physiological flexibility and emotional stability [5], thereby posing challenges for physiotherapists aiming to optimize both physical and psychophysiological recovery.

Breathing-based interventions have therefore become increasingly prominent in clinical and rehabilitation settings. Techniques such as slow breathing, HRV biofeedback, nasal strips, and mobile applications providing guided breathing exercises are used to correct dysfunctional patterns and enhance parasympathetic activity [6]. Among these, HRV biofeedback is particularly notable, as it enables individuals to monitor HRV in real time and regulate it through controlled breathing. Evidence suggest that HRV biofeedback can significantly reduce anxiety and stress, improve sleep quality, and increase HRV metrics [7,8].

Despite the growing interest in individual techniques, research remains limited on the combined effects of multi-component breathing interventions—particularly those integrating biofeedback with supportive tools—on HRV, respiratory function, and stress in individuals with dysfunctional breathing. Addressing this gap is important for physiotherapy, as such interventions may offer an evidence-based, non-pharmacological strategy for improving functional outcomes and quality of life.

The aim of this study was to evaluate the effectiveness of a comprehensive breathing intervention in individuals exhibiting signs of dysfunctional breathing. Specifically, the study examined the effects of multiple therapeutic components—including breathing exercises, nasal strips, guided mobile applications, and HRV biofeedback—on HRV parameters, respiratory function, and perceived stress. By focusing on these outcomes, this work contributes to the evidence base supporting physiotherapy-led breathing interventions and their role in promoting both rehabilitation and psychophysiological health.

## 2. Research Methodology

### 2.1. Research Methods

#### 2.1.1. Trial Design

This study was designed as a randomized, parallel-group exploratory trial with a 1:1 allocation ratio and conducted within a superiority framework. All trial procedures, outcomes, and analyses followed the original study protocol, with no post hoc modifications. The trial was carried out in a community fitness club in Klaipeda, Lithuania. All assessments (HRV, respiratory function, and stress measures) were conducted in a controlled environment within the same facility to ensure consistency of testing conditions.

The random allocation sequence was generated using a computer-based random number generator. To ensure allocation concealment and minimize selection bias, group assignments were placed into sequentially numbered, opaque, sealed envelopes prepared by a researcher who are not involved in participant recruitment or data collection. The envelopes were identical, tamper-proof, and stored securely. After a participant provided informed consent and met eligibility criteria, the next envelope in sequence was opened by the study coordinator, revealing the assigned group. This procedure ensured that the allocation sequence remained concealed until the moment of intervention assignment.

#### 2.1.2. Details of Patient or Public Involvement

Patients or members of the public were not directly involved in the design, conduct, reporting, or dissemination of this trial. The decision not to include patient or public involvement was based on the exploratory and methodological nature of the study, which primarily aimed to evaluate the physiological and psychological effects of breathing interventions under controlled conditions. Nevertheless, participants were fully informed about the procedures, potential risks, and anticipated benefits, and their feedback during the intervention period was monitored to ensure safety and adherence.

#### 2.1.3. Participant Characteristics and Sample Size

From the initial pool of 20 potential participants, 14 women aged 35–45 years who met the inclusion criteria and had no comorbidities were included in the final analysis. The age range of 35–45 years was selected to obtain a demographically homogeneous sample and to reduce age-related variability in autonomic regulation, respiratory function, and hormonal fluctuations. This approach facilitated clearer detection of intervention effects within the context of a pilot trial.

Participants were recruited through convenience sampling at a fitness club, where both men and women were eligible to participate. The final sample consisted exclusively of women by chance, as no male volunteers meeting the eligibility criteria enrolled during the recruitment period.

Participants were randomly assigned to either the control or intervention group using 1:1 allocation ratio. Both groups completed a 6-week program of guided breathing exercises using the iBreathe mobile application (version 3.1.2; iOS/Android) as the standard intervention, while the intervention group additionally used mouth tape during sleep.

The study was conducted as a pilot trial; therefore, no formal sample size calculation was performed. All 14 randomized participants completed both the baseline and post-interventions assessments. Since no data were missing, no imputation procedures were required, and all analyses were conducted on complete case data.

#### 2.1.4. Eligibility Criteria for Participants

Eligible participants were women aged 35–45 years, non-smokers, with no comorbidities and demonstrating signs of dysfunctional breathing as assessed by Hi-Lo test and breath-hold test. Exclusion criteria included the presence of comorbidities, any chronic medical conditions affecting respiratory or autonomic function, or current psychological or pharmacological treatment for stress or anxiety.

No specific eligibility criteria were applied to study sites or to individuals delivering the interventions, as the program consisted of self-administered breathing exercises guided by a mobile application and nocturnal mouth taping, neither of which requiring no specialist involvement.

#### 2.1.5. Blinding Procedure

Due to the nature of the intervention guided breathing exercises with or without mouth taping—blinding of participants and care providers could not feasible. The use of mouth tape during sleep made the intervention visibly distinguishable, preventing the possibility of participant blinding.

To minimize potential bias in measurement and analysis, outcome assessors and data analysts were blinded to group allocation. All data files were coded, and group labels remained concealed until the completion of statistical analyses. Because the interventions differed clearly in both appearance and procedure, no additional steps to simulate similarity between groups were implemented.

### 2.2. Testing Procedures

Physiological and psychological parameters were evaluated before and after the 6-week intervention period to assess its effectiveness. All testing sessions were conducted under similar conditions and at approximately the same time of day, to minimize circadian influences on physiological outcomes. The assessment protocol included objective measurements and validated self-report instruments.

#### 2.2.1. Heart Rate Variability (HRV)

HRV served as a primary indicator of autonomic nervous system regulation, particularly parasympathetic (vagal) activity. HRV was measured using the Polar H10 chest strap (Polar Electro Oy, Kempele, Finland) [9], a device widely recognized for its accuracy and reliability in capturing interbeat intervals (IBIs). Data were collected via the (version 5.6.1), which calculated both time-domain and frequency-domain parameters, including:

RMSSD (Root Mean Square of Successive Differences): a time-domain measure reflecting short-term HRV and parasympathetic activity.

HF (High Frequency) Power Component: a frequency-domain measure representing vagal modulation of heart rate associated with respiration (0.15–0.40 Hz).

#### 2.2.2. Respiratory Rate

Respiratory rate was assessed as an indicator of breathing pattern efficiency. While seated in a relaxed position, participants’ breathing frequency was counted manually over one minute. This measurement was repeated three times and the mean value was used for analysis. To improve accuracy, all assessments were conducted by the same trained researcher, and participants rested quietly for several minutes beforehand to stabilize breathing. A lower respiratory rate typically interpreted as a marker of improved respiratory efficiency and autonomic balance.

#### 2.2.3. Hencho Test (Breath-Holding Time)

Breath-holding time was used to evaluate respiratory control and tolerance to carbon dioxide accumulation. Participants took a normal inhalation and exhalation, then held their breath after a passive exhalation. The duration (in seconds) from the start of the breath-hold to the first involuntary urge to breathe was recorded. The Hencho test reflects functional breathing reserve and CO_2_ tolerance rather than functional residual capacity.

#### 2.2.4. Perceived Stress Scale (PSS-10)

Psychological stress was assessed using the 10-item Perceived Stress Scale developed by Cohen et al. [9]. The PSS-10 measures how unpredictable, uncontrollable and stressful participants perceive their life to have been over the past month. Items are rated on a 5-point Likert scale, yielding a total score from 0 to 40 with higher scores indicating greater perceived stress.

#### 2.2.5. Visual Analogue Scale (VAS) for Stress

Emotional stress during the testing period was measured using a 10-cm Visual Analogue Scale. The left endpoint represented minimal stress, and the right endpoint represented maximal stress. Participants placed a vertical mark on the line corresponding to their current level of stress. The score was determined by measuring the distance (in centimeters) from the starting point; each 10-cm line corresponds to 100%, with 1 mm representing 1% [10].

Assessments were conducted following standardized procedures to ensure consistency. Standardized instructions were provided before each measurement to reduce variability and potential bias.

#### 2.2.6. Statistical Methods

Data analysis was performed using IBM SPSS Statistics 24.0. Descriptive statistics (mean ± SD) were calculated for all variables. The Shapiro–Wilk test was used to assess the normality of distributions. Given the small sample size and the presence of several non-normally distributed variables, non-parametric tests were applied. Within-group differences (pre- vs. post-intervention) were evaluated using the Wilcoxon signed-rank test, while between-group differences were assessed using the Mann–Whitney U test. A *p*-value of <0.05 was considered statistically significant. Effect sizes were calculated using Cohen’s d.

Adverse events (harms) were monitored descriptively, and no statistical comparisons were required.

### 2.3. Intervention Procedure

Participants were recruited between February and April 2025. Follow-up assessments for benefits and potential harms were completed immediately after the 6-week intervention period. The intervention program lasted six consecutive weeks, during which participants in both the intervention and control groups followed a structured breathing protocol designed to enhance respiratory function and autonomic regulation.

#### 2.3.1. Breathing Exercises

All participants, regardless of group assignment, were instructed to perform guided breathing sessions twice daily using the iBreathe mobile application. The app provided auditory and visual cues to support consistent pacing and technique. Each daily routine included of the following components:

#### 2.3.2. Box Breathing (5 min)

This technique involves equal-duration phases of inhalation, breath-holding, exhalation, and post-exhalation hold (e.g., 4–4–4–4 s). Box breathing is known to activate the parasympathetic nervous system and contribute to improved focus and stress regulation [11].

#### 2.3.3. LSD (Long Slow Deep) Breathing (10 min)

This method emphasizes slow, controlled breaths—typically 6–8 breaths per minute—to promote vagal tone and respiratory efficiency. It is particularly effective in reducing breathing rate and increasing heart rate variability (HRV).

Participants were encouraged to complete both sessions daily—ideally once in the morning and once in the evening—in a calm environment while seated comfortably. To monitor adherence, they maintained a daily log, and weekly check-ins via phone or text were conducted to reinforce compliance.

#### 2.3.4. Additional Component for the Intervention Group

Participants in the intervention group were additionally instructed to use mouth tape during sleep for the duration of the six-week program. This component was introduced to promote nasal breathing at night, which is hypothesized to improve oxygen exchange efficiency, reduce sympathetic nervous system activation, and support more coherent breathing patterns. Commercially available hypoallergenic mouth tape was provided, along with written instructions and a demonstration of proper and safe application. Participants were advised to discontinue use if they experienced discomfort, sleep disturbances, or respiratory difficulty.

#### 2.3.5. Testing and Monitoring

All participants completed assessments at week 0 and post-intervention assessments at week 6. The testing protocol included physiological measures (HRV, respiratory rate, and Hencho test) and psychological measures (PSS-10, VAS). All assessments were conducted in a controlled environment and scheduled at similar times of day for each participant to minimize variability related to diurnal fluctuations in physiological parameters.

To ensure protocol adherence, participants received printed instructions and ongoing support throughout the study. Although objective adherence monitoring (e.g., wearable tracking devices), self-reported compliance indicated a high level of engagement in both groups.

#### 2.3.6. Harms

Potential harms—such as discomfort, sleep disturbance or respiratory difficulties—were monitored through weekly check-ins and participant self-report. Participants were instructed to discontinue intervention if any issues occurred.

### 2.4. Research Ethics

This study was conducted in accordance with the ethical principles of the Declaration of Helsinki. All procedures were developed and implemented to ensure the safety, well-being, and rights of the participants.

Ethical approval was obtained from Bioethics Committee of the Faculty of Health Sciences at Klaipėda State University of Applied Sciences. The study protocol, informed consent form, and all supporting materials were reviewed to confirm compliance with institutional and international ethical standards for research involving human participants.

All participants were fully informed about the aims, procedures, and potential risks and benefits of the study. Written informed consent form was obtained prior to enrollment. Participants were informed of their right to withdraw from the study at any time without penalty and without affecting their access to services or support.

To maintain confidentiality, all personal data were anonymized. Data were stored in password-protected digital files accessible only to the research team. No identifying information was used during data analysis, reporting, or publication.

The study involved non-invasive interventions (guided breathing exercises and nocturnal mouth taping) that posed minimal risk. Participant safety was monitored through weekly contact was to assess well-being and gather feedback. Participants in the intervention group received specific instruction on the safe application of mouth tape during sleep and were advised to discontinue its use immediately if they experienced discomfort, breathing difficulties, or sleep disturbances.

## 3. Results

### 3.1. Participant Flow and Data Completeness

All randomized participants (*n* = 14) completed both baseline and post-intervention assessments. Participants were analyzed in the groups to which they were originally assigned (intervention: *n* = 7; control: *n* = 7). No participants were withdrawn after randomization, and no missing data were recorded. Consequently, no imputation procedures, subgroup analyses, or sensitivity analyses were required. All analyses were conducted according to the predefined primary and secondary outcomes specified in the study protocol.

### 3.2. Demographic Statistics

A total of 14 participants were included in the final analysis, with 7 allocated to the intervention group and 7 to the control group. All participants were female and between 35 and 45 years of age, resulting in a demographically homogeneous sample. Random allocation ensured an even distribution between the groups.

The mean age of the intervention group was 38.57 ± 3.26 years, whereas the control group had a mean age of 39.29 ± 3.50 years. Statistical comparison using the Mann–Whitney U test showed no significant age difference between the groups (U = 21.5, *p* = 0.653), indicating effective randomization and baseline comparability.

None of the participants reported comorbidities or chronic medical conditions that could influence respiratory or autonomic function, and all were non-smokers.

Descriptive demographic characteristics are presented in Table 1.

### 3.3. Adverse Events

No adverse effects were reported during the study period.

### 3.4. Changes in Autonomic Nervous System Activity Based on HRV

Average heart rate variability (HRV) in the intervention group increased from 40.14 ± 5.43 at baseline to 45.29 ± 6.97 after the intervention. Although the mean change (+5.14 ± 8.45) indicated an improvement, it did not reach statistical significance (Z = −1.612; *p* = 0.107). In the control group, HRV increased from 53.29 ± 8.48 to 58.14 ± 6.07 (mean change: +4.86 ± 8.63), which was also not statistically significant (Z = −1.270; *p* = 0.204). A between-group comparison of the HRV change revealed no significant difference (U = 24; *p* = 0.949). These findings are summarized in Table 2.

RMSSD in the intervention group increased from 14.93 ± 4.58 ms to 16.11 ± 5.19 ms, with a mean difference of +1.18 ± 1.75 ms; however, this change was not statistically significant (Z = −1.352; *p* = 0.176). In the control group, RMSSD decreased slightly from 35.79 ± 17.63 ms to 34.52 ± 11.02 ms (mean change: −1.28 ± 9.58 ms), which was also non-significant. Between-group comparisons confirmed no significant difference in RMSSD change (U = 20; *p* = 0.565). These results are presented in Table 2.

The high-frequency (HF) component of HRV showed a statistically significant increase in the intervention group, rising from 0.26 ± 0.08 Hz to 0.28 ± 0.08 Hz (Z = −2.366; *p* = 0.018). In the control group, HF decreased slightly from 0.29 ± 0.09 Hz to 0.27 ± 0.11 Hz, a non-significant change (Z = −0.338; *p* = 0.735). The between-group comparison of HF changes did not reveal a significant difference (U = 18; *p* = 0.406). Full results are provided in Table 2.

### 3.5. Respiratory Function Outcomes

To evaluate respiratory system adaptation, two key functional parameters were analyzed: respiratory rate and Hench test duration. These measures provided insight into resting breathing efficiency and the functional respiratory reserve, particularly the body’s capacity to tolerate and retain carbon dioxide.

At baseline, the intervention group had an average respiratory rate of 21.00 ± 3.92 breaths per minute, which decreased to 17.43 ± 2.64 breaths per minute after six weeks. This reduction was statistically significant (Z = −1.992; *p* = 0.046). The control group also demonstrated a significant decrease, from 20.57 ± 2.23 to 16.00 ± 3.00 breaths per minute (Z = −2.388; *p* = 0.017).

However, the between-group comparison of respiratory changes did not reach statistical significance (U = 20; *p* = 0.561). These findings are presented in Table 3.

### 3.6. Hench Test

In the intervention group, Hench test duration increased significantly from 17.71 ± 4.22 s to 20.70 ± 4.07 s (Z = −2.366; *p* = 0.018), indicating improved breath-holding capacity. The control group showed only a small, non-significant increase, from 20.37 ± 1.36 s to 20.65 ± 1.53 s (Z = −0.507; *p* = 0.612).

The between-group comparison revealed a statistically significant difference favoring the intervention group (U = 7; *p* = 0.025), indicating that the intervention produced a more notable enhancement in respiratory reserve. Full results are presented in Table 3.

### 3.7. Subjective Perceived Stress Outcomes

In the intervention group, the mean PSS-10 score decreased significantly from 22.14 ± 4.98 to 14.43 ± 4.58 (Z = −2.375; *p* = 0.018), representing a mean reduction of 7.71 ± 3.68 points. Before the intervention, five participants reported moderate stress and two reported high stress; after the intervention, four reported moderate stress and three reported low stress (Z = −2.236; *p* = 0.025).

In the control group, the PSS−10 score also showed a significant reduction, decreasing from 23.14 ± 3.63 to 15.29 ± 3.15 (Z = −2.379; *p* = 0.017), with a mean decrease of 7.86 ± 2.54 points. At baseline, six participants reported moderate stress and one reported high stress; after the intervention, four reported moderate and three low stress (Z = −2.000; *p* = 0.046).

However, the between-group comparison of changes in PSS-10 scores revealed no statistically significant difference (U = 24; *p* = 0.949). These outcomes are summarized in Table 4.

### 3.8. Visual Analogue Scale (VAS)

In the intervention group, the mean VAS score decreased from 55.71 ± 19.88% to 42.86 ± 4.88%, a non-significant reduction of 12.86 ± 17.04% (Z = −1.604; *p* = 0.109). Similarly, the control group showed a non-significant decrease from 58.57 ± 9.00% to 47.14 ± 4.88% (Z = −1.841; *p* = 0.066), with a mean reduction of 11.43 ± 12.15%.

The between-group comparison of VAS changes was also non-significant (U = 24.5; *p* = 0.999). Prior to the intervention, 71.43% of participants reported high stress and 28.57% moderate stress. Post-intervention, 57.14% reported moderate stress and 42.86% low stress. Detailed results are presented in Table 4.

## 4. Discussion

The results of this study show positive outcomes in both the intervention and control groups, particularly improvements in respiratory rate and reductions in perceived stress. Although the overall increase in HRV within the intervention group did not reach statistical significance, the significant improvement in the HF component suggest enhanced parasympathetic nervous system activity. This aligns with previous research indicating that breathing exercises, especially those incorporating elements of biofeedback, can facilitate autonomic balance [11]. These results are relevant for physiotherapy practice, where interventions targeting autonomic regulation are increasingly integrated into rehabilitation strategies.

The significant decrease in respiratory rate observed across both groups supports earlier evidence that slow and conscious breathing enhances ventilatory efficiency and stimulates vagal pathways [12]. Notably, only the intervention group demonstrated a significant increase in Hencho test duration, indicating improved breath-holding capacity and respiratory control. This improvement may be associated with the introduction of mouth taping during sleep, which promotes nasal breathing and has been linked to the more efficient respiratory mechanics [13]. From a physiotherapy perspective, these findings highlight the potential value of adjunctive tools for optimizing functional breathing retraining.

Several physiological mechanisms may help explain these improvements. Slow, regulated breathing increases vagal tone, enhances baroreflex sensitivity, and stabilizes autonomic oscillations. which collectively contribute to improvements in HRV and subjective stress. Together, these mechanisms contribute to improved HRV and reductions in subjective stress, providing a plausible biological explanation for the observed changes in autonomic function and emotional well-being.

Reductions in perceived stress, as measured by the PSS, were significant in both groups, further reinforcing the therapeutic value of guided breathing for emotional regulation [5]. While VAS scores did not reach statistical significance, the downward trend suggests clinically meaningful benefits. These findings are consistent with previous studies by Jerath et al. [3] and Shaffer [5], which highlight the psychological and physiological benefits of breathing-based interventions. In the context of physiotherapy, where stress management is increasingly recognized as an integral component of holistic rehabilitation, the current results offer additional support for incorporating structured breathing interventions into clinical practice [14,15].

Overall, the results of this study suggest that structured breathing interventions—particularly when combined with supportive strategies such as mouth taping—may improve both physiological and psychological parameters in women with dysfunctional breathing. However, the small sample size limits the strength and generalizability of these conclusions. Larger trials are needed to confirm these preliminary findings and to establish clear, evidence-based guidelines for clinical application.

### Strength and Limitations

This pilot study has several limitations that should be considered. First, the age range (35–45 years) was intentionally selected to reduce age-related variability; however, a broader range (e.g., 25–45 years) could have been included. Future studies should consider wider age groups to enhance generalizability. Future studies should recruit more diverse samples to improve external validity. Second, the small sample size and short intervention duration constrain the ability to draw conclusions about long-term effects or sustained changes in breathing patterns. Future studies with larger and more diverse samples may benefit from incorporating patient perspectives when shaping research questions, developing intervention components, and planning dissemination strategies.

Adherence to the intervention—particularly the use of mouth tape—was based on self-report, which may have introduced reporting bias. Additionally, anthropometric variables such as height, weight, and BMI, as well as occupational information, were not collected in this pilot study, reducing the ability to fully characterize the sample and compare it with broader populations. Collecting these variables in future research would improve the external validity and contextual interpretation of results.

Although assessments were scheduled at similar times of day to reduce diurnal variation, individual differences in sleep patterns (e.g., irregular schedules, late-night work, or reduced sleep duration) may have influenced physiological outcomes. Another limitation involves the use of the iBreathe mobile application, a commercially available wellness tool rather than a regulated medical device. Its instructional content and algorithms have not undergone formal external validation by regulatory authorities, which may affect the standardization and reproducibility of app-based interventions. Incorporating patient perspectives into the development of research questions and intervention design may also strengthen future studies and enhance their relevance to clinical practice.

Despite these limitations, the study also possesses notable strengths. It employed a randomized controlled design; used objective physiological and functional outcome measures; and evaluated a novel combination of breathing interventions with supportive adjunctive strategies. These methodological strengths provide a valuable foundation for future large-scale trials and contribute meaningful preliminary evidence to physiotherapy and complementary rehabilitation practice. Breathing interventions may represent low-cost, accessible strategies to enhance autonomic balance and stress regulation in both community and rehabilitation settings, supporting wider public health initiatives aimed at improving self-regulation and preventive care.

## 5. Conclusions

This pilot study suggests that structured breathing interventions may contribute to improvements in respiratory efficiency and stress regulation among individuals with dysfunctional breathing. Although preliminary, the findings indicate that guided breathing practice—with or without adjunctive strategies such as mouth taping—can be incorporated into physiotherapy as a simple, non-pharmacological approach to enhancing autonomic balance and breathing control. Further research involving larger and more diverse samples is needed to confirm these effects and to determine the broader clinical applicability of these methods.

### Implications for Physiotherapy Practice

This study highlights several implications for physiotherapy and rehabilitation:

Integration into practice: Structured breathing interventions—particularly those delivered through mobile applications—can be feasibly incorporated into physiotherapy programs to address dysfunctional breathing patterns and support holistic patient care.

Adjunctive strategies: The inclusion of simple and low-cost supportive tools, such as mouth taping, may enhance the effectiveness of breathing retraining by improving HRV and respiratory control beyond what guided exercises alone can achieve.

Holistic outcomes: Improvements in Hencho test performance and reductions in perceived stress demonstrate the dual impact of breathing interventions on both physiological function and psychological resilience. This aligns with the holistic framework increasingly emphasized within physiotherapy practice.

Clinical application: Despite the limited scale of this pilot study, the findings provide a rationale for physiotherapists to incorporate breathing-based interventions into rehabilitation, wellness, and stress management programs. Future research is needed establish long-term effectiveness, determine optimal intervention protocols, and support evidence-based clinical guidelines.

Clinical relevance statement: Structured breathing interventions, supplemented by simple adjuncts such as mouth taping, may offer physiotherapists an accessible and low-cost approach to improving respiratory function, autonomic regulation, and stress management in individuals with dysfunctional breathing.

## Figures and Tables

**Table 1:** Demographic Characteristics of Participants.

Indicator	Intervention Group (M ± SD)	Control Group (M ± SD)	*p*-Value
Age (years)	38.57 ± 3.26	39.29 ± 3.50	0.653

Note. M—mean; SD—standard deviation.

**Table 2:** Changes in Heart Rate Variability (HRV) and Respiratory Function.

Variable	Group	Pre (M ± SD)	Post (M ± SD)	*p* Value	Cohen’s d
HRV (ms)	Intervention	40.14 ± 5.43	45.29 ± 6.97	0.107	0.82
HRV (ms)	Control	53.29 ± 8.48	58.14 ± 6.07	0.204	0.66
RMSSD (ms)	Intervention	14.93 ± 4.58	16.11 ± 5.19	0.176	0.51
RMSSD (ms)	Control	35.79 ± 17.63	34.52 ± 11.02	0.753	0.08
HF (Hz)	Intervention	0.26 ± 0.08	0.28 ± 0.08	0.018 *	0.75
HF (Hz)	Control	0.29 ± 0.09	0.27 ± 0.11	0.735	0.21

Notes: M—mean; SD—standard deviation; * statistically significant (*p* < 0.05). *p* values calculated with Wilcoxon signed-rank test; Cohen’s d calculated for paired samples.

**Table 3:** Respiratory Function Outcomes.

Indicator	Group	Pre-Intervention (M ± SD)	Post-Intervention (M ± SD)	*p*-Value (Within Group)	Cohen’s d
Respiratory Rate (breaths/min)	Intervention	21.00 ± 3.92	17.43 ± 2.64	0.046 *	0.96
Respiratory Rate (breaths/min)	Control	20.57 ± 2.23	16.00 ± 3.00	0.017 *	1.18
Hencho Test (s)	Intervention	17.71 ± 4.22	20.70 ± 4.07	0.018 *	0.71
Hencho Test (s)	Control	20.37 ± 1.36	20.65 ± 1.53	0.612	0.19

Note. Values are presented as means ± standard deviations. * Statistically significant at *p* < 0.05 (Wilcoxon signed-rank test). Cohen’s d effect sizes are interpreted as: 0.2 = small, 0.5 = medium, 0.8 = large.

**Table 4:** Changes in Subjective Perceived Stress Indicators Before and After the 6-Week Intervention.

Indicator	Before Intervention Intervention Group	After Intervention Intervention Group	p ^1^ Intervention	Cohen’s d Intervention	Before Intervention Control Group	After Intervention Control Group	p ^1^ Control	Cohen’s d Control
PSS (Perceived Stress Scale)	22.14 ± 4.98	14.43 ± 4.58	0.018 *	1.69	23.14 ± 3.63	15.29 ± 3.15	0.017 *	2.52
VAS, %	55.71 ± 19.88	42.86 ± 4.88	0.109	0.82	58.57 ± 9.00	47.14 ± 4.88	0.066	1.47

^1^ Wilcoxon signed-rank test. * *p* < 0.05—statistically significant. Cohen’s d: 0.2—small effect; 0.5—medium effect; ≥0.8—large effect.

## Data Availability

The raw data supporting the conclusions of this article will be made available by the authors on request.
